# Pea3 transcription factor promotes neurite outgrowth

**DOI:** 10.3389/fnmol.2014.00059

**Published:** 2014-06-26

**Authors:** Basak Kandemir, Berrak Caglayan, Barbara Hausott, Burcu Erdogan, Ugur Dag, Ozlem Demir, Melis S. Sogut, Lars Klimaschewski, Isil A. Kurnaz

**Affiliations:** ^1^Molecular Neurobiology Laboratory, Department of Genetics and Bioengineering, Yeditepe UniversityIstanbul, Turkey; ^2^Division of Neuroanatomy, Innsbruck Medical UniversityInnsbruck, Austria

**Keywords:** Pea3, neuron, axonal outgrowth, phosphorylation, neurofilaments

## Abstract

Pea3 subfamily of E–twenty six transcription factors consist of three major -exhibit branching morphogenesis, the function of Pea3 family in nervous system development and regeneration is only beginning to unfold. In this study, we provide evidence that Pea3 can directs neurite extension and axonal outgrowth in different model systems, and that Serine 90 is important for this function. We have also identified *neurofilament-L* and *neurofilament-M* as two putative novel targets for Pea3.

## INTRODUCTION

PEA3 subfamily of ETS (E–twenty six) domain transcription factors includes PEA3 (Polyoma enhancer activator 3)/E1AF (E1A enhancer binding protein)/ETV4 (Xin et al., 1992; Higashino et al., 1993), ER81/ETV1 (Monte et al., 1995), and ETS related molecule (ERM)/ETV5 in mammals (Laudet et al., 1999). Characteristically, ETS domain factors are involved in regulation of diverse sets of gene expression during tissue patterning and lineage commitment (Anderson et al., 1999; Chotteau-Lelievre et al., 2001), particularly in tissues that show branching morphogenesis such as lung and kidneys (Liu et al., 2003), as well as in metastatic tumors. Members of the PEA3 subfamily have been shown to be expressed in the developing nervous system of mouse from E9.5 to birth (Lin et al., 1998; Chotteau-Lelievre et al., 2001). Strong *erm* expression was seen in the caudal region, for example, with restricted expression in the cephalic region, prospective midbrain and ventral forebrain (Firnberg and Neubuser, 2002). Expression of *er81* in various mouse tissues of embryonic origin was less clear, with a_very faint signal being visible only by E9.5 at the frontonasal and branchial arch regions (Chotteau-Lelievre et al., 1997). In adults, ER81 transcript was found to be present in a variety of tissues, including heart, brain, lung, liver, pancreas, spleen, testis, and intestines (Monte et al., 1995). Pea3 was found to be expressed in the neural crest in zebrafish (Brown et al., 1998), and in the developing mouse embryo *pea3* expression was similar to that of *erm*, both genes being strongly expressed in posterior neural plate (Chotteau-Lelievre et al., 2001), while in adult mice Pea3 RNA is most abundant in the brain and the spinal cord (Xin et al., 1992; Laing et al., 2000), and was shown to mediate FGF signaling (Znosko et al., 2010). Furthermore, regulation of Pea3 through FGF18 signaling was shown to be important for the laminar positioning of developing neocortex (Hasegawa et al., 2004). However, ER81 was not found to be regulated by FGF in *Xenopus*, indicating a divergence in signal response of Pea3 family members (Roussigne and Blader, 2006). More recently, Pea3 was shown to be upregulated in DRGs upon NGF signaling during target innervation (Fontanet et al., 2013).

In concert with their pattern of expression, PEA3 family members have been shown to have a role in formation of functional neuron circuitry, both in chicken and mice (Ghosh and Kolodkin, 1998; Lin et al., 1998; Ladle and Frank, 2002). Particularly striking is the finding that in forming functional neuron circuits at the spinal cord level, PEA3 family members are selectively expressed in specific classes of motor neurons and corresponding muscle afferent sensory neurons at limb levels of the spinal cord in chick; namely, Pea3 positive sensory afferents and motor neurons form on circuit, while ER81 positive sensory afferents and motor neurons form a different circuit, indicating a selectivity (Arber et al., 2000). ER81 *loss-of-function* mutants have provided supporting evidence for its role in the establishment of sensory-motor circuits at the limb level (Arber et al., 2000). Pea3 knock-out mice, however, did not seem to have any major effects, except for ejaculation dysfunction in males, although it is suspected that the infertility of *pea3*^-^^/^^-^ males may have an underlying neuronal basis, particularly since Pea3 is expressed in specific bundles of motor neurons that innervate limb muscles and in afferent sensory neurons of these same muscles and it is conceivable that Pea3 is also expressed in neurons that innervate the penis (Laing et al., 2000). Unfortunately, since members of the PEA3 family of transcription factors have overlapping expression patterns, the full picture of their function in the organism may not be revealed until double or triple knockouts are established.

E–twenty six domain proteins have been shown to be targets for signaling pathways, particularly the MAPK pathway, during development (rev. Wasylyk et al., 1998; Sharrocks, 2001). ERM and Pea3 were implicated in nasal development in response to FGF signaling (Firnberg and Neubuser, 2002). In spite of all these pieces of data on the function of Pea3 subfamily at various levels of nervous system development, and its interaction with various upstream signals such as FGF, NGF, or GDNF, detailed molecular mechanisms of upstream signaling and downstream transcriptional targets are still far from understood. Most of the studies on Pea3 are carried out with respect to breast tumorigenesis, and several targets such as matrix metalloproteases have been identified with respect to metastasis (de Launoit et al., 2006), however how exactly Pea3 is involved in neuronal differentiation has remained unclear.

In this study, we wished to address if Pea3 was capable of differentiation of neuronal model cells, such as PC12, SH-SY5Y, or NSC-34, and even in adult DRG cells, and if so which target genes it may be regulating. We demonstrate that Pea3 can indeed induce neurite extension in these various systems, indicating that it may prove a useful target towards neuroregeneration, and we present evidence that MAPK signaling may be important for this process, as a transcriptionally inactive Serine90-to-Alanine mutant of Pea3 is incapable of differentiating NSC-34 cells *in vitro*. We further identify two presumptive novel targets of Pea3 to be *neurofilament-L* and *neurofilament-M*.

## MATERIALS AND METHODS

### PLASMIDS

Mammalian expression plasmids for wildtype mPea3 were kind gifts of Yvan de Launoit. pCDNA3 VP16 was constructed by cloning the acidic activation domain of VP16 transcription factor into the NcoI - Xba I sites of pCDNA3 vector. Pea3VP16 plasmid was constructed by cloning PCR amplification product of mouse Pea3 sequence into HindIII - XhoI sites of pCDNA3-VP16 plasmid. pCDNA3-Pea3 was constructed by cloning the PCR amplification product of mouse PEA3 sequence into the HindIII - XbaI sites of pCDNA3 vector. pEGFP-C3 was used in co-transfection experiments. Pea33-Luciferase reporter construct, where 5 × Pea3 optimal sites upstream of the TK minimal promoter drives the expression of the luciferase reporter gene.

Human Neurofilament M (NFM; promoter ID 39810) and Neurofilament L (NFL; promoter ID 41145) promoter sequences were retrieved from Cold Spring Harbor Laboratory “Transcriptional Regulatory Element Database” tool^1^, and were analyzed for putative Pea3 protein binding sites with ALGGEN PROMO^2^, a virtual laboratory to study transcription factor binding sites. Binding sites within the restricted NFM and NFL promoters were named as “*ets*” and sites with highest Pea3 binding affinity (with the dissimilarity rate below 7%) were selected to mutate or delete to study the *trans*-activation capacity of Pea3.

Cloning of wild type NFL and NFM promoters were cloned and mutations of the putative Pea3 binding regions on these promoters were introduced by using primers in **Table 1**. Amplification products of NFM and NFL promoters (both spanning a region from -953 to +64 for *hNF-L* and from -654 to +10 for *hNF-M*) were cloned into *KpnI* - *HindIII* sites of pGL2 - Basic vector (Promega).

**Table 1:** List of primers used for cloning promoter luciferase constructs and the mutants thereof.

*hNFL*-Luc forward	5′-ACGAGACGGTACCGTGCTGCGGTTGGTGG-3′
*hNFL*-Luc reverse	5′-ACGAGACAAGCTTTGGGAGCCCGGAGAGAG-3′
*hNFM*-Luc forward	5′-ACGAGACGGTACCGAAAAGGATCTCCGAGG-3′
*hNFM*-Luc reverse	5′-ACGAGACAAGCTTGCTGTCACAGCGTTCT-3′
*NFL*Δ *1*-Luc forward	5′-AATCAGGAGAAGATGAATTGCA-3′
*NFL*Δ *1*-Luc reverse	5′-TGCAATTCATCTTCTCCTGATT-3′
*NFL*Δ *2*-Luc forward	5′-GGCAACTTACCAAGTGTCACG-3′
*NFL*Δ *2*-Luc Reverse	5′-CGTGACACTTGGTAAGTTGCC-3′
*NFM*Δ-Luc Forward	5′-AGACGGTACCGAAGAGGGGCCAAA-3′
*NFM*Δ-Luc Reverse	5′-ACGAGACAAGCTTGCTGTCACAGCGTTCT-3′

Serine residues on mouse Pea3 protein that are potential targets for Proline-directed phosphorylation were mutated by two-step PCR-based site directed mutagenesis method. Essentially, forward and reverse primers were designed to replace the codon for serine with codon for either alanine or glutamic acid (**Table 2**). In the first step of PCR, 100 ng pcDNA3-mPea3 was mixed with the 1.25 U *Pfu* DNA polymerase (Promega), *Pfu* buffer (Promega) to final concentration 1X, dNTP mix to final concentration 200 μM each, and forward and reverse primers (at varied concentrations) with mutant codons were paired with ultimate reverse and ultimate forward primers that constraints wild type mouse Pea3 coding sequence, respectively, run at the 56°C annealing temperature. Amplicons from first step of PCR were run at 46°C for six cycles with 10× PrimeStar buffer (Takara) to final concentration 1×, PrimeStar DNA polymerase (Takara) to final concentration 1.25 U and PrimeStar (Takara) dNTP tofinal concentration 200 μM each to initiate annealing of overlapping mutant regions. To obtain the full length Pea3 gene coding sequence, second step of PCR was run at 56°C for 30 cycles with the previous reaction mix completed with ultimate forward and reverse primers and additional PrimeStar DNA polymerase and PrimeStar buffer.

**Table 2:** List of primers used for cloning phosphor-mutants of mPea3.

mPea3 S90A Forward primer	5′GCT TTC CAT GCC CCC ACC3′
mPea3 S90A Reverse primer	5′GGT GGG GGC ATG GAA AGC3′
mPea3 S90E Forward primer	5′GCT TTC CAT GAA CCC ACC3′
mPea3 S90E Reverse primer	5′GGT GGG TTC ATG GAA AGC3′

*Sites that are mutated are underlined.*

Resultant PCR products were then cloned into the *Hind III*- *XhoI* sites of pcDNA3 and pCMV3-Tag-6. Bacterial transformation was done for the amplification of resultant plasmid constructs and for further validation analysis. The validation of the cloning was done by the colony PCR where positive bacterial colonies were directly used as a PCR template, then restriction enzyme digestion was done with *Hind III*-*XhoI* for further confirmation of the presence of Pea3 coding sequence. Plasmid clones from positive bacterial colonies were then purified for sequence analysis for final confirmation of presence of Pea3 sequence with desired mutation (data not shown). Pea3 phospho-mutant plasmids were further analyzed for proper gene expression and translation (data not shown).

### CELL CULTURE

PC12 pheochromacytoma cells were maintained in DMEM supplemented with 10% Horse serum and 5% Fetal Bovine serum in the presence of antibiotics, penicillin and streptomycin, and L-glutamine. Differentiation experiments were performed in collagen-coated plates, unless otherwise stated (Collagen Type IV, Sigma C0543). Transient transfections were carried out with Effectene reagent (Qiagen 301425), following manufacturer’s instructions. Nerve growth factor (NGF 2.5 S, Sigma N6009) was used commonly at 50-100 ng/ml. Epidermal growth factor (EGF, Sigma E4127) was used at 100 nM. Basic fibroblast growth factor (bFGF) and Insulin-like Growth Factor-1 (IGF-1) were used at final concentrations of 20 ng/ml and 100 ng/ml, respectively.

Mouse Motor Neuron (NSC-34) cell line and SH-SY5Y neuroblastoma cell line were grown in the high glucose DMEM (Gibco, 1129855) supplemented with 10% Fetal Bovine serum in the presence of penicillin, streptomycin, L-Glutamine and amphotericin B (Biological Industries, 03-033-1B) and primocin (Invivogen, ant-pm-1). Differentiation experiments were performed in collagen-coated plates (Collagen I, Gibco A10483-01). SH-SY5Y cells (10^6^ cells/dish) were treated with 0.3 μM of aphidicolin (Sigma, 10797) in serum-free medium. After 24 h, the cells were treated with 10 μM retinoic acid (RA, Sigma R2625) to induce neuronal differentiation. Cells were collected on day 0, 1, and 3 for further analysis as required.

Dorsal root ganglia (DRG) from adult rats were dissected, collected in RPMI medium with antibiotic-antimycotic and treated with collagenase (5000 U/ml) for 60 min followed by 0.25% trypsin/EDTA for 15 min. They were then transferred to RPMI medium containing 10% horse serum and 5% fetal bovine serum and gently triturated by 5-10 passages through a fire-polished Pasteur pipette. About 20 softened DRG were co-transfected with 3 μg Pea3 and 1 μg DsRed or with 1 μg DsRed alone in 100 μl Rat Neuron Nucleofector^®^ solution (Lonza, Austria) using the Amaxa Nucleofector (program O-03). Transfected neurons were plated onto poly-D-lysine/laminin pre-coated dishes. Cultures were maintained in RPMI medium with B27 supplement (Gibco Invitrogen) and antibiotic-antimycotic at 37°C in a humidified atmosphere with 5% CO_2_. No approval for extracting tissue from dead animals was required. The DRGs used for cell culture were obtained from young adult rats immediately after exsanguinations. This procedure did not involve an animal experiment. Hence no Ethical Committee Approval was necessary.

### IMMUNOFLUORESCENCE

For immunofluorescence, the cells (PC12 or NSC-34) were commonly seeded on 12-well plates. For differentiation experiments, cells were commonly co-transfected with GFP plasmid and live cells were routinely scored at 48 and 96 h after transfection under inverted microscope (Nikon ECLIPSE TE200). The expression plasmid-to-GFP ratio was commonly 3:1. Differentiation was scored as the percentage of neurite-bearing cells (at least two cell body length was taken as the cut-off point) among the GFP-expressing cell population. At least three different fields per well, and at least two different wells per transfection were scored, and average and standard deviation calculated using MS Excel software. The results were represented as the average of duplicate wells (three fields per well) in at least two independent experiments.

### REVERSE TRANSCRIPTION POLYMERASE CHAIN REACTION

Total cytoplasmic RNA was prepared using commercial kits (RNAeasy kit, Qiagen, cat. no. 74104, or GeneJET RNA Purification kit, Thermo cat. no. #K0731) using manufacturer’s instructions. 1 μg (M-Mu-LV-Rtase, Roche) or 500 μg (New England BioLabs E6300L) RNA was used for each first strand cDNA synthesis reaction, as per instructions of the mentioned manufacturer, using random primers (Boehringer Mannheim). The amount of cDNA used was standardized using GAPDH and linear range determined. Typically the RT-PCR reactions were performed using 10–50 ng cDNA template in 25 μl reaction with BioTaq polymerase or 20 μl reaction with iTaq polymerase, at 50–64°C, as required, for 26–30 cycles. GAPDH primers (ADS1036/1037) were 5′ AGACAAGCTTCAGAGCCACCCGGGACC and 5′AGACTCTAGATCGGAGTCAACGGATTTGG; Pea3-specific primers (ADS1046/1047) were 5′ GACAAGCTTCGCCTACG ACTCAGATGTC and 5′ GACTCTAGAAGCTCCAATCCCTTCCTGC; *Neurofilament-L* primers were 5′ CAGTCTGGAGAACCTCGACC and 5′ TTCCAGGACCTTGTTCTGCT; *Neurofilament-M* primers were 5′AGGCATCGCACATCACGGTGGAG and GGATATTGTGATTGGGGGTCG. The products were resolved in 2.5 % Nu-Sieve agarose gels and were analyzed using QuantiOne imaging software (BioRad).

### LUCIFERASE REPORTER ASSAYS

HEK293 and SH-SY5Y cells were grown in 1 g/ml DMEM (Sigma) supplemented with 10% fetal bovine serum (GIBCO-Invitrogen), and 1% antimycotic-antibiotic (GIBCO-Invitrogen). 24 h prior to transfection, cells were seeded into 24-well plates with the density of 50 thousand cells per well. Plasmid transfections of cells were performed using 1.5 μl of TransFast^TM^ (Invitrogen) for reporter assays with NFM and NFL promoter reporter constructs. Essentially, plasmids were transfected per well in the following amounts: 100 ng Renilla Luciferase (Promega), 200 ng reporter construct (NFM*-Luc* or NFL-*Luc*) and wild type Pea3 expression plasmid with varying concentrations as indicated in the text (usually 5, 25, 50, 100, 150, and 200 ng, completed up to 200 ng DNA in total with pCMV empty vector). After 48 h, cells were harvested and lysed for 15 min with Luciferase Cell Culture Lysis 5× Reagent (Promega) diluted to 1× with PBS. Thirty microliter of cell lysate triplicate for each sample was transferred to the white opaque luminometer microtiter 96-well plate and mixed first with the 30 μl Dual-Glo^®^ Luciferase Substrate to measure luciferase activity from promoter of interest and later 30 μl Dual-Glo^®^ Stop & Glo^®^ Substrate to measure luciferase activity from internal control *Renilla* Luciferase. Luciferase activity was measured with Luminoskan Ascent (ThermoLab Systems) for 10 s for each substrate.

### CHROMATIN IMMUNOPRECIPITATION (CHIP)

Chromatin Immunoprecipitation (ChIP) assay was carried out with EZ-ChIP^TM^ ChIP Kit (Catalog # 17-371) according to the manufacturer’s instructions. Essentially, DNA was sheared enzymatically with the micrococcal nuclease. Some of the sheared DNA was saved as input, and rest of the sample was precipitated using 30 μl of anti-FLAG M2 affinity resin (Sigma) that was previously resuspended in Triton lysis buffer to precipitate 100 μl of Flag-tagged Pea3 protein that is bound to sheared DNA. Pea3 protein bound DNA was then eluted by reverse crosslink reaction in high salt environment enhanced with heat treatment. DNA in the samples was purified by using PureLink PCR Purification Kit (Invitrogen). Purified DNA from both input and ChIP samples were then detected by q-PCR (NFM *ets-1* spanning region forward primer: 5′-AAGGGCAGGGTGAACTGGACT-3′; NFM *ets-1* spanning region, reverse primer: 5′-TTCTTTAGCCTTCTACCCTCTTATCCTC- 3′). Percent Input was calculated so as to normalize qPCR data, and essentially signal intensities obtained from the ChIP samples were divided by signal intensities obtained from the Input sample.

## RESULTS

### EXOGENOUS Pea3 CAN ENHANCE NEURITE FORMATION IN PC12 CELLS

**FIGURE 1:**
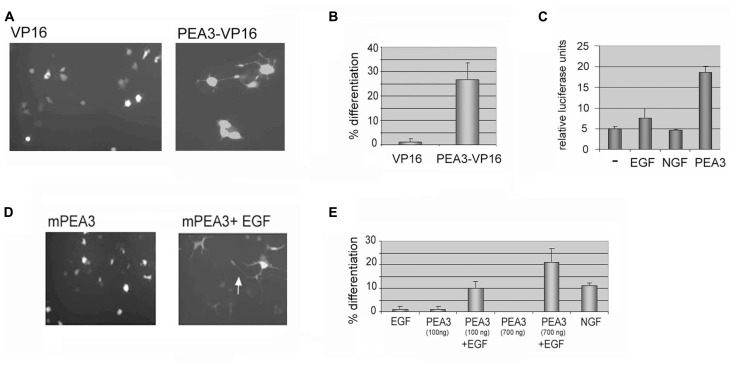
**Either a constitutively active Pea3-VP16 fusion or Pea3 induced with EGF can induce neurite formation in PC12 cells. (A)** A VP16 fusion of mouse Pea3 leads to neurite formation in PC12 cells. Cells were transfected with 500–1 μg of GFP expressing plasmid in 1:3 ratio with either a control plasmid expressing VP16 acidic activation domain alone, or a fusion of Pea3 to VP16 activation domain, and analyzed by fluorescence microscopy. A typical micrograph is shown. **(B)** The cells were scored for % neurite formation among the GFP-expressing population. (Two different DNA preparations were used for Pea3VP16 plasmid; data shown for only one of these.) **(C)** Luciferase assays in response to NGF, EGF, and transfected Pea3 on SRE-Luciferase reporter construct; average of at least three independent experiments. **(D)** A typical micrograph of cells transfected with 500–1 μg of GFP expressing plasmid in 1:3 ratio with either a control plasmid expressing pCDNA3 (not shown), pCDNA3 Pea3, or pCDNA3 Pea3 in combination with EGF as described, and analyzed by fluorescence microscopy. The arrow shows axon-like processes several cell body length. **(E)** A graph summarizing the scoring for differentiation experiment in **(D)**. The experiment was repeated with two different DNA preparations for pCDNA3 Pea3, and at least 3 fields per well were counted. The control plasmid pCDNA3 yielded no differentiation (not shown), while NGF in a similar time scale (2–4 days after transfection) resulted in around 12% differentiation (not shown).

In order to study the effects of Pea3 directly in differentiation, a chimeric protein with full length mouse Pea3 and the potent acidic activation domain of VP16 transactivator of the Herpes Simplex Virus (HSV) was constructed. The fusion protein was confirmed to activate from an artificial luciferase reporter (data not shown). When exogenous Pea3-VP16 fusion was expressed in PC12 cells, it induced neurite formation within 2 days, regardless of collagen-coating (**Figure 1A**). The percentage of neurite-bearing cells was significantly increase, up to around 25%, when Pea3-VP16 was transfected to PC12 cells (**Figure 1B**). Expression from an SRE-Luciferase reporter in PC12 cells was not greatly altered upon stimulation of PC12 cells that were serum starved for 36 h and stimulated with either EGF or NGF overnight, whereas cells transfected with exogenous mPea3 showed significant increase in luciferase reporter activity (**Figure 1C**), indicating that even mPea3 without any VP16 fusion was transcriptionally active in this context.

Although a constitutively active Pea3-VP16 fusion protein is useful in studying the effects of an activated Pea3, it does not represent a physiologically relevant situation. Therefore, PC12 cells were next transfected with mouse Pea3, either in the presence or absence of Epidermal Growth Factor (EGF). In contrast to Nerve Growth Factor (NGF), which results in sustained MAPK activation and neuronal differentiation, EGF is known to lead to proliferation in these cells through transient activation of the MAPK pathway (Marshall, 1995; Aksan and Kurnaz, 2003). Although EGF is not sufficient to drive differentiation of PC12 cells on its own, in combination with exogenous mPea3 it was seen to enhance long axonal projections (**Figures 1D,E**).

### EXOGENOUS Pea3 CAN ENHANCE BOTH FGF-INDUCED AND IGF-1-INDUCED AXONAL GROWTH IN PRIMARY DRG NEURONS

**FIGURE 2:**
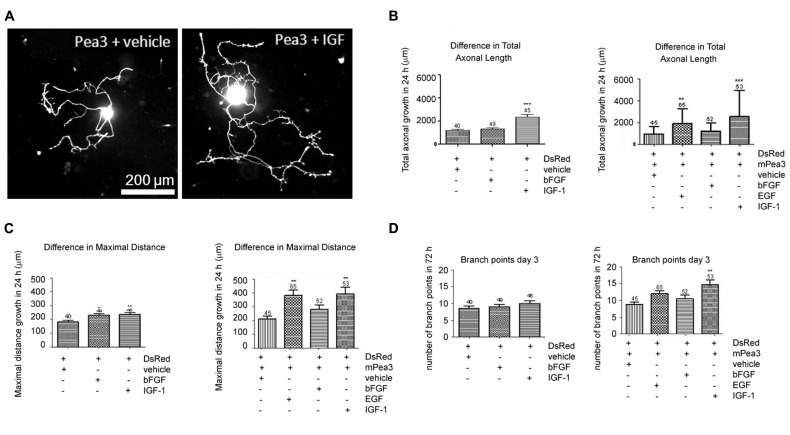
**Adult DRG neurons co-transfected with Pea3 expression vector and DsRed and stimulated with bFGF, EGF, or IGF-1. (A)** representative images of DRG neurons co-transfected with Pea3 and DsRed, stimulated with IGF-1; **(B)** total axonal growth in adult DRG neurons transfected with DsRed alone (left panel; IGF-1 and bFGF compared with control, using one way ANOVA test with Tukey’s post test, **p < 0.01, ***p < 0.001), or co-transfected with DsRed and mPea3, in the presence or absence of growth factors (right panel; EGF, IGF-1, and bFGF compared with control, using one-way ANOVA test with Tukey’s post test, **p < 0.01, ***p < 0.001); **(C)** difference in maximal axonal outgrowth normalized to control cells transfected with DsRed alone (left panel; IGF-1 and bFGF compared with control; using one way ANOVA test with Tukey’s post test, *p < 0.01, **p < 0.001), or co-transfected with DsRed and mPea3, in the presence or absence of growth factors (right panel; EGF, IGF-1, and bFGF compared with control, using one-way ANOVA test with Tukey’s post test, **p < 0.01, ***p < 0.001); **(D)** number of branch points in adult DRG neurons transfected with DsRed alone (left panel; IGF-1 and bFGF compared with control; using one way ANOVA test with Tukey’s post test, **p < 0.01, ***p < 0.001), or co-transfected with DsRed and mPea3, in the presence or absence of growth factors (right panel; EGF, IGF-1 and bFGF compared with control, using one-way ANOVA test with Tukey’s post test, **p < 0.01, ***p < 0.001).

In order to further analyze whether Pea3 would have the same effect on axonal outgrowth in adult neurons, we have studied adult DRG cultures transfected with mPea3 expression vectors, in combination with EGF, bFGF, and IGF-1. It was observed that IGF-1 alone could cause a significant increase in total axonal growth (around 2000 microns), while bFGF alone could not (around only 1000 microns) in DRGs transfected with DsRed plasmid alone (**Figures 2A,B**). When these neurons are co-transfected with DsRed and mPea3 expression vector and then stimulated, it was observed that while stimulation of mPea3-expressing cells with bFGF did not cause a significant axonal growth, stimulation with EGF caused a nearly twofold increase (although variability was high between different DRG cultures due to transfection efficiency), and IGF-1 on mPea3-expressing cells caused a nearly threefold increase in total axonal growth (**Figure 2B**).

The results were similar in maximal distance growth analyses, with EGF and IGF-1 stimulations equally enhancing total growth when mPea3 was overexpressed in cells, as compared to DsRed alone (**Figure 2C**). In terms of number of branches, however, presence or absence of exogenous mPea3 did not have a significant effect; there was only a slight enhancement in number of branch points when mPea3-expressing cells were stimulated with EGF or IGF-1 (**Figure 2D**).

### WILDTYPE Pea3 CAN INDUCE DIFFERENTIATION IN NSC-34 MOTOR NEURON CELL LINE, BUT A PHOSPHORYLATION MUTANT CANNOT

**FIGURE 3:**
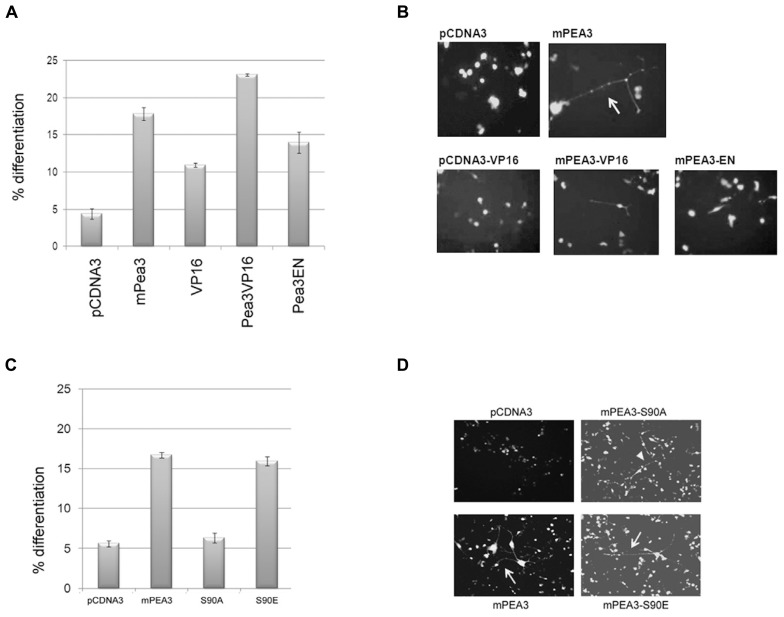
**Pea3 can enhance axonal outgrowth in NSC-34 motor neuron cell lines. (A)** Percent differentiation as scored by the number of green fluorescent cells with axonal outgrowth longer than 2 cell body length versus total GFP-expressing cells (two sets of duplicates are shown, at least four different fields are scored per well); **(B)** representative images of NSC-34 cells transfected with either empty pCDNA3 vector, pCDNA3-mPea3, pCDNA3-VP16, pCDNA3-mPea3-VP16 or pCDNA3-mPea3-EN; **(C)** Percent differentiation by Pea3 phosphorylation mutants as scored by the number of green fluorescent cells with axonal outgrowth longer than two cell body length versus total GFP-expressing cells (two sets of duplicates are shown, at least four different fields are scored per well); **(D)** representative images of NSC-34 cells transfected with either empty pCDNA3 vector, wildtype pCDNA3-mPea3, or the phosphorylation mutants pCDNA3-mPea3-S90A, pCDNA3-mPea3-S90E.

NSC-34 is a commercially available motor neuron cell line (produced through the fusion of embryonic mouse spinal cord cells with neuroblastoma cells); they are capable of differentiation as readily monitored by extensive axonal outgrowth, and are readily transfected, making them valuable developmental models. When these model cells were co-transfected with an empty expression vector, pCDNA3, and pEGFP, there was only background spontaneous differentiation in around 5% of the GFP-positive cells (**Figure 3A**), while transfection of Pea3 along with pEGFP plasmid lead to differentiation of around 15-20 % of the GFP-positive cells. pCDNA3-VP16/pEGFP co-transfection resulted in around 10% differentiation, whereas pCDNA3-Pea3VP16/pEGFP co-transfection resulted in well over 20% differentiation in GFP-positive cells (**Figure 3A**). Interestingly, Pea3-EN, a dominant negative form of Pea3 in fusion with the repression domain of Engrailed transcription factor, also showed some enhanced differentiation (around 15%), although this was lower than differentiation induced by either Pea3 or Pea3VP16. Representative images of NSC-34 differentiation experiments are shown in **Figure 3B**. While the motor neuron-derived cells normally appear round in morphology, in Pea3- or Pea3-VP16-transfected cells rather extensive axon-like neurite outgrowths of more than 2 cell body length are readily observed and scored as differentiation.

Since phosphor-specific Pea3 antibodies are not commercially available, we have generated phosphor-mutants of Pea3; particularly Serine 90 was shown to be important for transcriptional activity, as in both PC12 and SH-SY5Y cells Pea3S90A mutants showed basal level of activity on SRE-Luciferase reporter, while Pea3S90E mutants showed even enhanced activity as compared to wildtype Pea3 (data not shown). When NSC-34 cells were transfected with the wildtype mPea3 expression plasmid, differentiation was observed to increase nearly threefold, around 15%, as compared to cells transfected with empty pCDNA3 plasmid, as in the previous assay (**Figure 3C**; compare to **Figure 3A**). Exogenous expression of a transcriptionally defective Pea3-S90A mutant resulted in basal level of differentiation, whereas that of a transcriptionally active Pea3-S90E mutant resulted in differentiation to the same level as wildtype Pea3 (**Figures 3C,D**).

### NEUROFILAMENT-L AND NEUROFILAMENT-M AS POTENTIAL TARGETS OF Pea3

Since in experimental model systems Pea3 overexpression lead to morphological changes similar to differentiation, the next question was identifying which target genes would be responsible for this transformation. The known transcriptional targets of Pea3 mostly include matrix metalloproteases, such as MMP-2 and MMP-9, identified in the context of breast or prostate cancer models. Therefore, in an attempt to identify more neurologically relevant targets of Pea3, we have initially started investigating genes that are correlated with neuronal differentiation and whose promoters contained putative Pea3-binding motifs.

**FIGURE 4:**
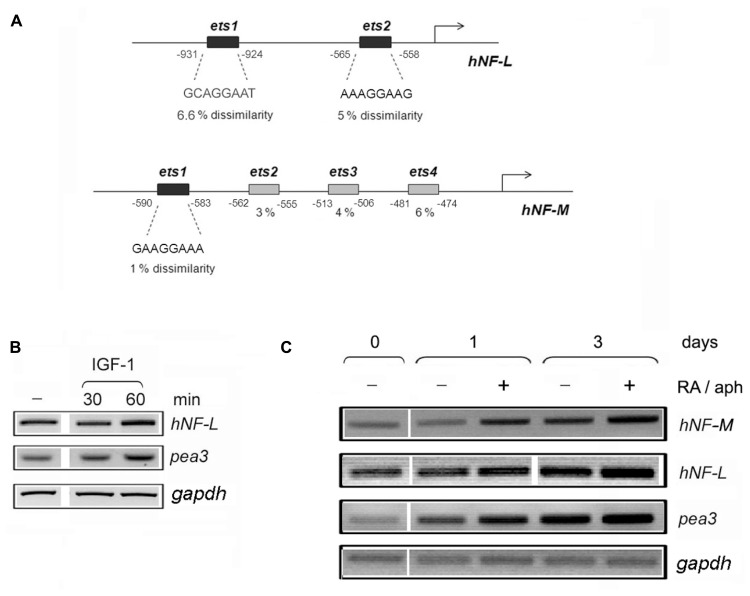
**Neurofilament-L and –M promoters contain several *ets* motifs with significant similarity to Pea3 consensus binding site. (A)** Human *Neurofilament-L* and *–M* promoters were analyzed by with Promo 3.0 (http://alggen.lsi.upc.es/cgi-bin/promo_v3/promo/promoinit.cgi?dirDB=TF_8.3), and dissimilarity rates (which indicate the variance between the binding motif of the transcription factor and the nucleotide sequence on the promoter with respect to predicted transcription start site, shown as percentage with respect to the binding matrices) are written below the predicted *ets* motifs; **(B)** the expression analysis of *NF-L* and *pea3* genes in SH-SY5Y cells stimulated with IGF-1 for 30 and 60 min; **(C)** the expression analysis of *NF-M, NF-L, pea3* and *gapdh* genes in SH-SY5Y cells before (day 0) or after (days 1 and 3) of stimulation in the presence (+) or absence (-) of Retinoic acid and aphidicolin (RA/aph) as described in Sections “Materials and Methods.”

Upon initial bioinformatic analysis, two neuron-specific promoters were particularly striking with respect to their putative binding motifs: neurofilament-L and neurofilament-M. The promoter sequences for these genes were obtained through the Transcriptional Regulatory Element Database (TRED) Cold Spring Harbor Laboratory^3^, and thereafter analyzed by ALGGEN PROMO^4^, a virtual laboratory for the study of transcription factor binding sites in DNA sequences, to identify potential Pea3 binding motifs. Human *Neurofilament-L (NF-L)* promoter contained only two Pea3-specific *ets* motifs, with 6.6 and 3.9% dissimilarity scores (**Figure 4A**; it should be noted that mouse *NF-L* promoter contains 4 *ets* motifs, two with 0% and two with 1% dissimilarity scores, and rat *NF-L* promoter contains 3 *ets* motifs, with 0, 1, and 6% dissimilarities; not shown). Human *NF-M* promoter, on the other hand, contained four *ets* motifs, only one of which shared significant homology to consensus Pea3-binding motif (1% dissimilarity; **Figure 4A**). The other three *ets* motifs in the human *NF-M* promoter indicated 3, 4, and 6 % dissimilarities; however when mouse and rat promoters were analyzed, rat promoter was found to have 4 *ets* motifs, with 0.4, 5, 5, and 1.7 % dissimilarities, and mouse promoter was found to have again 4 *ets* motifs with 6, 5, 3, and 1.7 % dissimilarities, data not shown). The *ets* motifs predicted on the *Neurofilament-H* promoter yielded much lower similarities (not shown), hence not included in this study. We have therefore proceeded with our analyses, focusing on the only 2 *ets* motifs in the *Neurofilament-L* promoter, and the highest scoring *ets* motif in the *Neurofilament-M* promoter, in order to estimate whether these promoters indeed would respond to Pea3-induced transcriptional regulation.

Our first approach was to confirm whether *Pea3/ETV4* message was indeed upregulated, albeit modestly, in human SH-SY5Y cells in response to stimulation with IGF-1 treatment, since IGF-1 caused the most prominent neurite extension in the DRG studies. To that end, we have serum starved SH-SY5Y overnight, and treated them with IGF-1 for 30 and 60 min. RT-PCR experiments confirmed a modest increase in *Pea3/ETV4* expression at 60 min, parallel to a comparable increase in *hNF-L* expression (**Figure 4B**). In a complementary experiment, we have differentiated SH-SY5Y cells in the presence of Retinoic Acid (RA) and aphidicolin, as described in Materials and Methods, for 3 days (**Figure 4C**). Pea3 expression levels gradually increase with retinoic acid and aphidicolin treatment even at Day 1 (close to 2- and 2.5-fold in the absence and presence of treatment, respectively; this slight increase even in the absence of treatment can be due to spontaneous differentiating cells due to crowding). This increase went up to around threefold in the absence and around 3.5-fold in the presence of treatment on day 3 (**Figure 4C**). When *NF-L* levels were similarly analyzed, a modest increase (around 1.2-fold) was detected upon stimulation with retinoic acid and aphidicolin treatment on day 1, which went up to 1.7-fold in the absence and 2.2-fold in the presence of treatment on day 3. Yet, due to already high basal levels of endogenous *NF-L* in cell lines, the most prominent change was observed in the level of *NF-M* expression, with twofold increase on day 1 and around fourfold increase on day 3 of treatment (**Figure 4C**).

**FIGURE 5:**
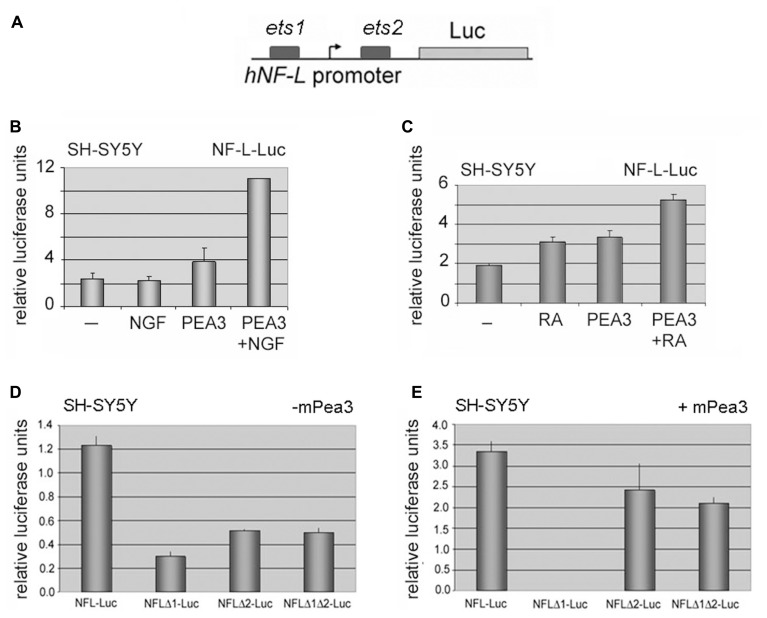
**Neurofilament-L as a potential target promoter for activation by Pea3. (A)** hNF-L promoter, which contains 2 major *ets* motifs, was cloned upstream of a luciferase reporter gene; **(B)** luciferase assays in SH-SY5Y human neuroblastoma cells transfected with or without mPea3, in the presence or absence of NGF stimulation; **(C)** luciferase assays in SH-SY5Y human neuroblastoma cells transfected with or without mPea3, in the presence or absence of retinoic acid (RA) stimulation; **(D,E)** SH-SY5Y cells are co-transfected with wild type NF-Lgene promoter driven luciferase reporter plasmid or with NF-L gene promoter which lacks putative Pea3 binding sites ets-1 (NFLΔ1-Luc) or ets-2 (NFLΔ2-Luc) or both (NFL Δ1Δ2-Luc). Relative luciferase activities were monitored in the presence **(D)** or absence **(E)** of 200ng of wild type Pea3. Luciferase activities represent reproducible three or four independent experiments, and data plotted for each condition were average of relative luciferase activity of triplicate samples.

In order to analyze whether this increase in *NF-L* and *NF-M* expression was a direct consequence of transcriptional regulation by Pea3, we have initially cloned the *hNF-L* promoter upstream of a luciferase coding sequence, as described in Section “Materials and Methods” (**Figure 5A**). When SH-SY5Y cells transfected with *hNFL*-Luc reporter were cultured in the presence or absence of NGF, a basal level of stimulation was observed; co-expression of Pea3 in these cells in the absence of stimulation resulted in modest increase in reporter activity, whereas stimulation with NGF in the presence of exogenous Pea3 resulted in more than twofold activation when compared to Pea3 alone (**Figure 5B**). A similar upregulation was observed when cells were stimulated with retinoic acid in addition to exogenous Pea3 expression (**Figure 5C**). To confirm that the *ets* motifs were indeed important for this regulation, we have then constructed *ets* motif deletion constructs, termed *NFL*Δ*1*, *NFL*Δ*2*, and *NFL*Δ*1*Δ*2*, referring to deletion of *ets1*, deletion of *ets2*, or deletion of both *ets1* and *ets2* motifs. In the absence of exogenous Pea3 transfection to the cells, deletion of either or both *ets* motifs resulted in loss of basal level activation from the *hNF-L* promoter (**Figure 5D**). However when these deletion constructs were co-transfected to SH-SY5Y cells together with Pea3 expression plasmid, deletion of *ets1* motif resulted in almost complete repression of reporter activity (**Figure 5E**), indicating that this motif is important for Pea3-induced transcription from this promoter, whereas deletion of *ets2* motif resulted in only a modest decrease, if at all; strangely, deletion of both *ets1* and *ets2* resulted in almost restoration of basal level activity, indicating that *ets2* may in fact be a repressive element of *NF-L* promoter.

**FIGURE 6:**
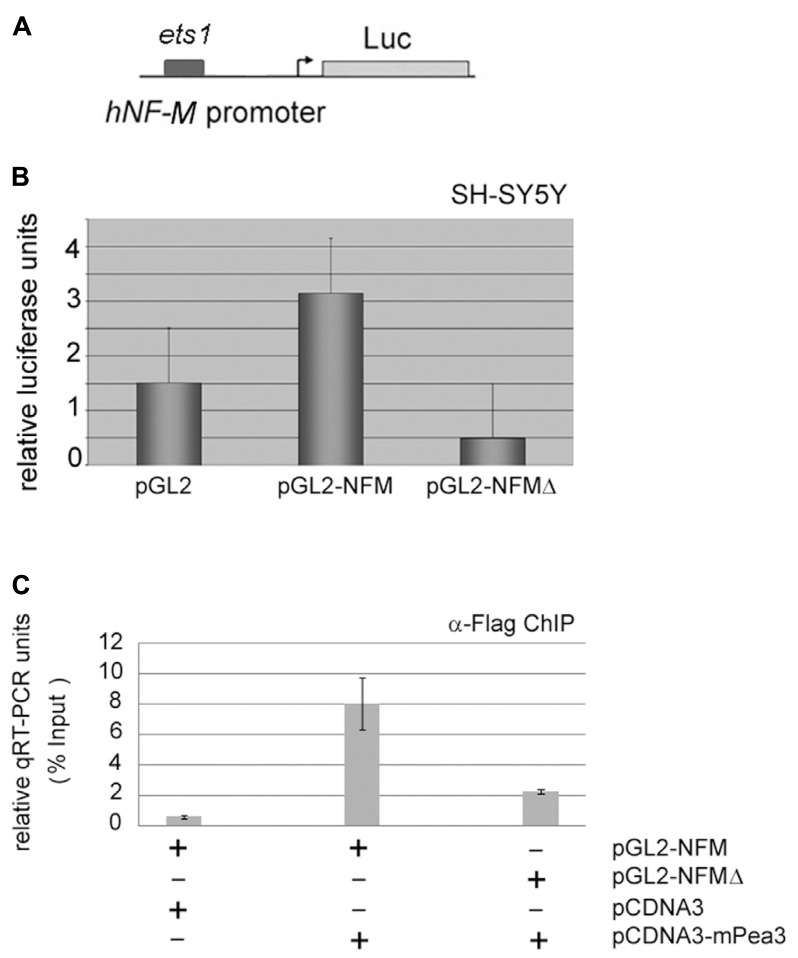
***Neurofilament-M* as a potential target promoter for activation by Pea3. (A)** hNF-M promoter, which contains 1 major *ets* motif, was cloned upstream of a luciferase reporter gene; **(B)** luciferase reporter gene expression assay with either wild type 200 ng of NFM promoter or NFM-Δ with deletion of putative Pea3 transcription factor binding site, in SH-SY5Y cells (luciferase activities represent reproducible three to four independent experiments and data plotted for each condition were average of relative luciferase activity of triplicate samples); **(C)** quantitative PCR results of chromatin immunoprecipitation (ChIP) assay, where Flag-tagged mPea3 expression plasmid was co-transfected into the HEK293 cells with or without NFM wild type and NFM-Δ luciferase reporter, subjected to chromatin precipitation with anti-Flag antibody and purified DNA material was amplified with NF-M gene promoter primers.****

When a similar analysis was carried out for the *hNF-M* promoter, which contains a single *ets* motif with significant homology to consensus Pea3 binding site (**Figure 6A**), it was observed that *hNF-M-*Luc reporter showed a nearly twofold activitation in response to Pea3 as compared to empty reporter, which was significantly repressed when *ets* motif was deleted (*NFM*Δ; **Figure 6B**). Since this highest-scoring *ets* motif appeared to be important for transcriptional activation, we have then analyzed Pea3 binding through ChIP assay: essentially, we have co-transfected SH-SY5Y cells with pCDNA3-Pea3-Flag expression plasmid along with either pGL2-NFM or pGL2-NFMΔ reporter plasmid, and immunoprecipitated Pea3 protein as described in Section “Materials and Methods,” thereafter amplified the *ets-*containing promoter region by PCR. A reaction was observed only when Pea3 and *hNFM*-Luc were co-transfected to the cells, but not the deletion reporter construct (**Figure 6C**), indicating Pea3 indeed could bind to this *ets* motif on the *hNFM* promoter.

## DISCUSSION

The expression profile of Pea3 family members in development is quite diverse, with earliest appearance reportedly at embryonic day E9.5 in brain regions, followed by Erm and Er81 expression in the lung at E10.5, in thymus at E12.5, urogenital tract at E13.5, cartilage at E14.5, and mammary tissue at E15.5, with all three proteins expressed in skeletal muscles, sensory, and motor neurons, and even in the colon (Maroulakou and Bowe, 2000). It was shown that Pea3 subfamily of ETS domain transcription factors are involved in a temporally regulated manner at later stages of nervous system development, in particular for normal sensory neuron differentiation and during branching (Lin et al., 1998; Haase et al., 2002; Hippenmeyer et al., 2005). Paratore et al. (2002) have shown that, unlike Pea3 and Er81, which are more important at later stages of development, Erm is involved at early neuronal differentiation of neural crest stem cells.

Different growth factors were shown to regulate Pea3 family members, for instance Fibroblast Growth Factor (FGF) was shown to regulate Pea3 proteins during development at various brain regions as well as retina (Weisinger et al., 2010), while GDNF, Met, HGF receptor was shown to play a role for recruitment of Pea3-positive neurons to motor neuron pools (Helmbacher et al., 2003). As stated above, Pea3 and Erm proteins were found to be expressed at late stages of lung development and is regulated by FGF signaling, however whether this is correlated with innervation with neurons (Liu et al., 2003), possibly in a manner similar to myogenic differentiation (although in myogenesis Pea3 was shown to be expressed in undifferentiated myoblasts at earlier stages of myogenic differentiation; Taylor et al., 1997), has not been clearly shown. Fontanet et al. (2013) have recently shown that Pea3 expression was induced in DRG neurons that are stimulated by NGF at the axon, indicating that this neurotrophin may indeed regulate Pea3 during target innervation.

In spite of many such studies emphasizing the importance of Pea3 subfamily proteins in various neuronal systems, targets of Pea3 transcriptional activity with respect to nervous system have not been fully understood. In *C. elegans* system ETS protein Ast-1 (axon steering defect-1) was shown to be responsible for dopaminergic neuron differentiation, and in the same system some of the major dopaminergic genes were shown to contain *ets* motifs as important regulatory elements (Flames and Hobert, 2009). Similar set of genes have not yet been identified for mammalian systems. So far, cadherin-8, ephrin receptor 4 (Ephr4) and semaphorin-3E were shown to be targets of Pea3 in neurons (Livet et al., 2002; Koo and Pfaff, 2002).

In our study, we have shown that transfection of Pea3 into various different neuronal model cells was sufficient to induce axon-like outgrowths, indicating that Pea3 could indeed be directly involved in regulation of neuronal maturation and/or differentiation in embryonic-derived motor neurons, but also in adult DRGs, implying it may have a role in neuroregeneration. We have further identified two potential novel targets for Pea3, namely, neurofilament-L and neurofilament-M. Furthermore, in a parallel ongoing microarray study, NF-L expression was found to be upregulated 1.87-fold in mPea3-transfected SH-SY5Y cells (manuscript in preparation). Our findings are supported by a study where FGF was shown to regulate Pea3 expression in the developing chick retina in a MAPK-dependent manner, with Pea3 being particularly expressed throughout retinal epithelium from stage 23 (McCabe et al., 2006). In this chick study, the authors have shown that Pea3 and neurofilament-M double-positive neurons were only present at the newly generated ganglion cell layer, and that FGF receptor inhibitor not only reduces Pea3 expression but also NF-M expression, confirming our findings in rat and human cell line models.

There are various different and parallel transcriptional, signaling, and guidance-related events involved in different aspects of neuronal development in the vertebrates (reviewed in Polleux et al., 2007), therefore the genes identified in our study as novel Pea3 targets are likely to be only the tip of an iceberg of transcriptional targets, that need to be identified for each Pea3 subfamily member separately in different neuronal systems. In this study we have only demonstrated the capacity of Pea3 to regulate neuronal axon formation, and have shown potential phosphorylation site Serine 90 to play an important part in this function. More detailed analyses are required for a detailed dynamics of Pea3-dependent transcriptional regulation in various aspects of nervous system components.

## Conflict of Interest Statement

The authors declare that the research was conducted in the absence of any commercial or financial relationships that could be construed as a potential conflict of interest.

## Footnotes

1 http://rulai.cshl.edu/cgi-bin/TRED/tred.cgi?process=home

2 http://alggen.lsi.upc.es/cgi-bin/promo_v3/promo/promoinit.cgi?dirDB=TF_8.3

3 http://rulai.cshl.edu/cgi-bin/TRED/tred.cgi?process=searchPromForm

4 http://alggen.lsi.upc.es/cgi-bin/promo_v3/promo/promoinit.cgi?dirDB=TF_8.3
